# X‐ray Crystallography and Vibrational Spectroscopy Reveal the Key Determinants of Biocatalytic Dihydrogen Cycling by [NiFe] Hydrogenases

**DOI:** 10.1002/anie.201908258

**Published:** 2019-10-25

**Authors:** Yulia Ilina, Christian Lorent, Sagie Katz, Jae‐Hun Jeoung, Seigo Shima, Marius Horch, Ingo Zebger, Holger Dobbek

**Affiliations:** ^1^ Institut für Biologie, Strukturbiologie/Biochemie Humboldt-Universität zu Berlin Philippstraße 13 10115 Berlin Germany; ^2^ Institut für Chemie Technische Universität Berlin Straße des 17. Juni 135 10623 Berlin Germany; ^3^ Max-Planck-Institut für Terrestrische Mikrobiologie Karl-von-Frisch-Str. 10 35043 Marburg Germany; ^4^ Department of Chemistry and York Biomedical Research Institute University of York Heslington York YO10 5DD UK

**Keywords:** biocatalysis, crystal structure, hydrogen activation, [NiFe] hydrogenase, vibrational spectroscopy

## Abstract

[NiFe] hydrogenases are complex model enzymes for the reversible cleavage of dihydrogen (H_2_). However, structural determinants of efficient H_2_ binding to their [NiFe] active site are not properly understood. Here, we present crystallographic and vibrational‐spectroscopic insights into the unexplored structure of the H_2_‐binding [NiFe] intermediate. Using an F_420_‐reducing [NiFe]‐hydrogenase from *Methanosarcina barkeri* as a model enzyme, we show that the protein backbone provides a strained chelating scaffold that tunes the [NiFe] active site for efficient H_2_ binding and conversion. The protein matrix also directs H_2_ diffusion to the [NiFe] site via two gas channels and allows the distribution of electrons between functional protomers through a subunit‐bridging FeS cluster. Our findings emphasize the relevance of an atypical Ni coordination, thereby providing a blueprint for the design of bio‐inspired H_2_‐conversion catalysts.

## Graphical abstract

**Where does the H_2_ go?** Using an F_420_‐reducing [NiFe]‐hydrogenase from *Methanosarcina barkeri* as a model enzyme, it is shown that the protein backbone provides a strained chelating scaffold that tunes the [NiFe] active site for efficient H_2_ binding and conversion. The protein matrix also directs H_2_ diffusion to the [NiFe] site via two gas channels and allows the distribution of electrons between functional protomers through a subunit‐bridging FeS cluster.

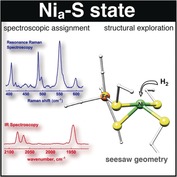

## Introduction

Mapping out strategies for future energy storage and conversion represents one of the central challenges of the 21^st^ century. Molecular hydrogen (H_2_) is an ideally clean fuel whose combustion releases large amounts of free energy but no greenhouse gases. To exploit it to its full potential, however, we require efficient and sustainable approaches for catalytic H_2_ cleavage and formation. [NiFe] hydrogenases are valuable model enzymes that catalyze H_2_ conversion at rates comparable to platinum electrodes by using a heterobimetallic active site containing cheap and earth‐abundant base metals only.^1^ Their rational utilization as biotechnological targets or blueprints for bio‐inspired catalysts, however, requires a thorough understanding of the structural and mechanistic determinants of their reactivity. Here, we employ a bidirectional F_420_‐reducing [NiFe] hydrogenase from the archaeon *Methanosarcina barkeri* MS (*Mb*FRH) as a unique model system to yield structural and spectroscopic insights into a scarcely explored reaction intermediate that is the initial target for H_2_ binding to the active site of the enzyme. The structure of this central catalytic species is analyzed in detail to explore its relevance for the mechanism and performance of [NiFe] hydrogenases.

## Results and Discussion

**Figure 1:**
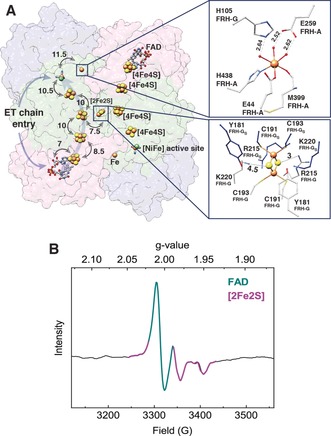
A) Reversible ET (indicated by arrows) between the FAD and the [NiFe] active site is enabled by a chain of [4Fe4S] clusters. The [NiFe] center could also exchange electrons with a mononuclear Fe site (upper inset), and the presence of a [2Fe2S] cluster (lower inset) allows electrons to commute between antiparallel ET chains of two heterotrimers. Distances given in Å; selected atoms and amino acids shown as spheres (Fe: orange, S: yellow, Ni: green) and sticks, respectively. B) EPR spectra of an *Mb*FRH solution recorded at 80 K with 1 mW microwave power and 9.3 GHz microwave frequency. Signals from FAD and a [2Fe2S] cluster are highlighted in dark cyan and violet, respectively.

The crystal structure of *Mb*FRH was refined using reflections up to *d*_min_=1.84 Å (Supporting Information, Table S1). Each asymmetric unit contains three subunits, FRH‐A, FRH‐B, and FRH‐G, and a total of four [4Fe4S] clusters as well as a flavin adenine dinucleotide (FAD) cofactor and the [NiFe] active site (detailed below). The FAD and the heterobimetallic [NiFe] center enable the redox conversion of the two substrates, coenzyme F_420_ and H_2_, respectively, while the [4Fe4S] clusters mediate intramolecular electron transfer (ET) between the two reaction sites (Figures 1 A and S1). These features are shared with the related [NiFe] hydrogenase from *Methanothermobacter marburgensis* (*Mm*), and both enzymes exhibit a dodecameric overall architecture in a spherical shape (Figure S1).^2^ Compared to the latter enzyme, however, *Mb*FRH contains two additional cofactors that are both ET‐accessible and solvent‐exposed: a [2Fe2S] cluster bridging two FRH‐G subunits (Figures 1 A and S2) and a mononuclear Fe site in FRH‐A (Figures 1 A and S3).

Based on element‐specific anomalous scattering, the single Fe ion has also been assigned in [NiFeSe] hydrogenases,^3^ while Ca^2+^ 4 or Mg^2+^ 5 ions are typically found at the same position in other [NiFe] hydrogenases (Table S2). The [2Fe2S] cluster likely contributes to the biological function of *Mb*FRH by enabling the distribution of electrons among individual protomers of the dodecameric enzyme. In line with this proposal, solution‐phase electron paramagnetic resonance (EPR) experiments reveal signals from FAD, [2Fe2S] clusters (Figures 1 B and S4), and [4Fe4S] clusters (Figure S4), corroborating the extended electron‐transfer pathway of native *Mb*FRH.

**Figure 2:**
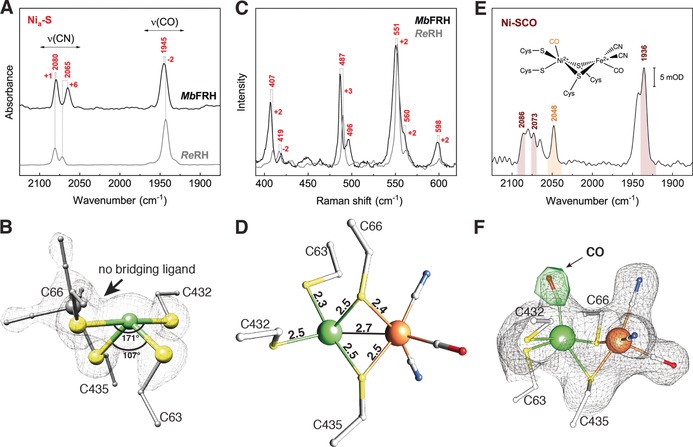
A) IR spectra of an *Mb*FRH single crystal (at 80 K, black) and a protein solution of *Re*RH (10 °C, gray). Spectra were normalized with respect to the CO stretching‐band intensity. B) Crystal structure of the [NiFe] active site, exhibiting a distorted seesaw coordination geometry of the Ni(Cys)_4_ moiety and a vacant coordination site between the Ni and Fe ions. The 2 *F*_obs_−*F*_calc_ electron density map after full refinement is shown as a gray mesh (1.8 σ). C) RR spectrum of an *Mb*FRH single crystal (black) compared to that of a protein solution of *Re*RH.11b RR spectra were recorded at 80 K using 568‐nm laser excitation and normalized with respect to the most intense signal at 551/553 cm^−1^. D) [NiFe] active‐site crystal structure. Selected interatomic distances are given in Å. E) IR absorbance spectrum of an *Mb*FRH single crystal, recorded at 80 K. Bands corresponding to the intrinsic, Fe‐bound diatomic ligands and the extrinsic, Ni‐bound CO of the Ni‐SCO redox‐structural state are highlighted in brown and orange, respectively. F) X‐ray structure of the CO‐inhibited [NiFe] active site. The 2 *F*_obs_−*F*_calc_ electron density map after full refinement (1 σ) and the residual *F*_obs_−*F*_calc_ map (5.5 σ) before CO‐modeling are shown as a gray mesh and green surface, respectively. Selected atoms and amino acid residues are shown as spheres (Ni in green, Fe in gray (B) and orange (D and F), S in yellow) and sticks, respectively.

In the following, we will focus on the [NiFe] active site of *Mb*FRH, which exhibits the consensus structural properties observed for other [NiFe] hydrogenases.^6^ Specifically, this heterobimetallic cofactor features two metal ions, Ni and Fe, that are coordinated by four strictly conserved cysteinate (Cys) residues and three Fe‐bound diatomic ligands. Based on infrared (IR) spectroscopic analyses, the latter constituents are generally assigned to one CO and two CN^−^ ligands.^7^ Consistently, one CO and two CN stretching bands can be observed in IR spectra of *Mb*FRH crystals, which confirms the presence of a standard set of inorganic ligands (Figure 2 A, black trace). Since these stretching vibrations are highly sensitive towards structural and electronic changes at the [NiFe] center,^8^ the observation of a single set of three IR absorption bands in the relevant spectral range also demonstrates that the active site resides in a homogenous redox‐structural state. In contrast to crystal structures of many other [NiFe] hydrogenases, no electron density can be detected in the third bridging position between the two metals for *Mb*FRH (Figure 2 B).6a–6c This excludes the presence of oxygen‐containing ligands that would be indicative of inactive enzyme residing, for example, in the Ni_u_‐A or Ni_r_‐B states.6a, 9 Notably, a vacant third bridging site between the two metals has long been assumed to be a key feature of Ni_a_‐S (also termed Ni‐SIa), the H_2_‐binding intermediate of [NiFe] hydrogenases.6a, 10 This assumption has been recently supported by spectroscopic analyses,^11^ but detailed structural insights into this central catalytic intermediate have been so far unavailable. In the following, we will shed light on these catalytic key aspects by using a joint approach of crystallographic analysis and vibrational spectroscopy.

While the presence or absence of bridging oxygen ligands can be clearly established from crystal‐structure analyses, H_2_‐derived (hydride) ligands are only observable in sub‐atomic‐resolution structures,^12^ and available crystal structures lacking detectable bridging ligands most likely represent hydride species.9e, 13 Since crystals of *Mb*FRH have been prepared in a mildly reducing atmosphere containing up to 5 % H_2_, the derived structural data could reflect Ni_a_‐S or a hydride species. Thus, before analyzing the active‐site structure of *Mb*FRH in more detail, we studied the underlying crystals using different vibrational‐spectroscopic techniques to firmly identify the [NiFe] redox‐structural state.

IR spectra of *Mb*FRH crystals reveal CO and CN stretching bands at 1945 and 2065/2080 cm^−1^, respectively, which resemble the IR fingerprint of the Ni_a_‐S state of several [NiFe] hydrogenases (Figure 2 A).6a In particular, these vibrational frequencies are close to those observed for the as‐isolated regulatory hydrogenase from *Ralstonia eutropha* (*Re*RH), which is a spectroscopically valuable yet structurally unexplored reference system for the Ni_a_‐S state.^11^ Based on this finding, contributions from the Ni_a_‐C hydride intermediate and its photo‐inducible congener, Ni_a_‐L, can be excluded since these catalytic species would give rise to higher and lower CO stretching frequencies, respectively.6a The fully reduced Ni_a_‐SR state, however, cannot be excluded on the basis of IR‐spectroscopic data alone since its dominating sub‐species exhibits an IR fingerprint similar to that of Ni_a_‐S in several [NiFe] hydrogenases.6a Thus, we next recorded resonance Raman (RR) spectra of *Mb*FRH crystals to probe Fe−CO and Fe−CN metal–ligand vibrations as structural markers of the [NiFe] active site (Figure 2 C).11b, 14 In these measurements, Ni_a_‐SR would be photo‐converted to Ni_a_‐L, while Ni_a_‐S would remain unaffected in terms of structural and electronic properties.11b, 14 Again, the obtained vibrational signature is very similar to that of the Ni_a_‐S state as observed for *Re*RH and a membrane‐bound hydrogenase from the same organism (*Re*MBH).11b, 14b In particular, a structurally sensitive vibrational mode with considerable Fe−CO stretching character can be detected at 596–598 cm^−1^, which excludes contributions from Ni_a_‐SR, since its photoproduct Ni_a_‐L would give rise to an Fe−CO stretching frequency above 600 cm^−1^.11b, 14a Moreover, the intensity of the RR spectrum was found to increase with the excitation wavelength of the Raman probe laser, which is in line with previous observations for Ni_a_‐S11b, 14 and contrary to expectations for reduced [NiFe] hydrogenases.11b

Finally, we also explored the effect of treating *Mb*FRH crystals with CO, a typical inhibitor of [NiFe] hydrogenases.6a, 15 We performed these experiments to check for interaction of CO with the [NiFe] active site, which is expected for Ni_a_‐S (yielding Ni‐SCO) but not for Ni_a_‐C or Ni_a_‐SR.15d Binding of extrinsic CO to the terminal vacant coordination site at the Ni ion of *Mb*FRH is evident from the electron density at this position (Figures 2 F and S6) and a high‐frequency CO stretching band at 2048 cm^−1^ in the corresponding IR spectrum (Figure 2 E), as also observed for other [NiFe] hydrogenases in the Ni‐SCO state.15a, 15c, 15d Both structural and spectroscopic data show that this inhibited species accumulated to at least 50 % (Figure S6), while the remainder can be assigned to the Ni_a_‐S parent state. In total, the above experiments show that the crystal structure of untreated *Mb*FRH reflects a homogenous Ni_a_‐S state, allowing a detailed analysis of this H_2_‐binding intermediate.

Structural and electronic properties of Ni_a_‐S have been proposed to be essential for efficient H_2_ binding and hydride formation in [NiFe] hydrogenases.11b, 16 In the following, we will revisit these proposals to evaluate their validity based on the crystal structure. While there is wide agreement regarding the overall catalytic mechanism of [NiFe] hydrogenases, details about the central steps of H_2_ binding and activation are, so far, elusive. In particular, the site of initial H_2_ binding is not known, and either of the two metal ions may be involved in the formation of a (side‐on) H_2_ σ‐bond complex from Ni_a_‐S. Experimental data on this first catalytic step are not yet available, but recent theoretical studies favor the Ni ion as the initial site of H_2_ binding.^16^, ^17^ According to these studies, the coordination geometry of this metal ion represents a key to the energetically favorable interaction of H_2_ with the [NiFe] active site. Specifically, a peculiar seesaw‐shaped geometry with *trans* S−Ni−S angles approaching 120° and 180° was postulated to be mandatory for thermodynamically favorable binding of H_2_ to Ni_a_‐S.^16^ In line with this proposal, the [NiFe] active site of *Mb*FRH exhibits *trans* S−Ni−S angles of 107° and 171°, thereby structurally confirming this unusual geometry of the Ni_a_‐S state (Figure 2 B). This finding also agrees with previous RR studies on *Re*RH,11b highlighting the merit of combining crystallographic, spectroscopic, and theoretical methods.

Notably, four‐coordinate Ni^II^ sites, as found in Ni_a_‐S, typically exhibit (distorted) square‐planar or tetrahedral coordination geometries. This indicates that the atypical seesaw geometry of this [NiFe] intermediate is dictated by the four‐cysteinate coordination pattern and the protein matrix of [NiFe] hydrogenases.^16^, ^18^ Remarkably, *trans* S−Ni−S angles in the Ni_a_‐S state of *Mb*FRH resemble those found in computationally optimized Ni_a_‐S geometries of previous theoretical studies (124° and 151°), but comparison with the underlying crystal‐structure‐derived starting geometries (109° and 166°) yields an even better agreement.^16^ This indicates that the magnitude and relevance of the structural constraints imposed by the protein matrix is even more pronounced than previously anticipated. Further evidence for the relevance of structural constraints comes from comparing the experimental Ni−Fe distance of Ni_a_‐S to values obtained in theoretical studies. Calculations on small‐ to medium‐size models typically report Ni−Fe distances of up to 3.3 Å,6a, 11b, 19 while models including larger parts of the protein matrix yield smaller values,^19^ close to those we observe in the crystal structure (2.7 Å; Figure 2 D). This indicates that the protein matrix compresses the Ni−Fe distance to values close to those observed for other catalytic intermediates, including hydride species, Ni_a_‐C and Ni_a_‐SR (2.6 Å),^12^ and presumed metal–H_2_ adducts (2.6–2.8 Å).^16^, 17b This effect likely minimizes structural reorganization during H_2_ turnover, thereby adding to the remarkable catalytic efficiency of [NiFe] hydrogenases.11b Additionally, a short Ni−Fe distance may also be relevant for metal–metal bond formation, as previously proposed for catalytic intermediates of [NiFe] hydrogenases.^20^ Remarkably, Ni−Fe distances obtained from large computational models only reproduce the experimental value observed for *Mb*FRH if a low‐spin (*S*=0) configuration of the [NiFe] active site is assumed in the calculations,^16^, ^19^ supporting a singlet ground state of Ni_a_‐S in *Mb*FRH.

**Figure 3:**
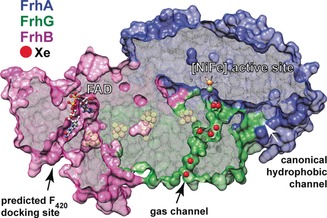
Seven Xe atoms (shown as red spheres) were detected within the noncanonical channel cutting through the FRH‐G subunit. FRH‐A, FRH‐B, and FRH‐G are represented by surfaces and colored navy blue, violet, and green, respectively.

To further explore the initial interaction of *Mb*FRH with H_2_, we also investigated possible intramolecular H_2_ transfer pathways. To this end, *Mb*FRH crystals were derivatized with xenon to explore hydrophobic gas channels connecting the exterior of the enzyme with the [NiFe] active site (Figures 3 and S7 A). These experiments revealed a second H_2_‐transfer channel that has not been observed in similar studies on other [NiFe] hydrogenases.^21^ Surprisingly, the unrelated [NiFeSe] hydrogenase from *Desulfovibrio vulgaris* Hildenborough features a similar hydrophobic tunnel (Figure S7B), indicating analogous developments in certain hydrogenases that operate bidirectionally in vivo.^22^ While this narrow channel appears obstructed in crystal structures of other [NiFe] hydrogenases (Figure S7 B),21a, 21b dynamically enhanced H_2_ transfer via this route remains as a far‐reaching possibility and a target for future studies.

## Conclusion

In the current account we have experimentally explored the unique structural properties of the H_2_‐binding catalytic intermediate of [NiFe] hydrogenases. Besides revealing additional electron and H_2_ pathways, the spectroscopically validated crystal structure of a pure Ni_a_‐S state unraveled two key determinants of efficient H_2_ cycling: a peculiar seesaw‐shaped coordination geometry of the Ni ion and a short Ni−Fe distance that is indicative of a low‐spin electronic ground state. Both structural observations contradict expectations for unconstrained low‐molecular‐weight transition‐metal compounds, thereby illustrating the central role of chelating ligand scaffolds and outer coordination layers in biocatalytic H_2_ cycling. These findings expand our understanding of [NiFe] hydrogenases and, thus, provide valuable guidance for the future design of bio‐inspired catalysts for H_2_‐based energy‐conversion approaches.

## Conflict of interest

The authors declare no conflict of interest.

## Supporting information

As a service to our authors and readers, this journal provides supporting information supplied by the authors. Such materials are peer reviewed and may be re‐organized for online delivery, but are not copy‐edited or typeset. Technical support issues arising from supporting information (other than missing files) should be addressed to the authors.

Supplementary
