# Supplementary material for: X‐ray Crystallography and Vibrational Spectroscopy Reveal the Key Determinants of Biocatalytic Dihydrogen Cycling by [NiFe] Hydrogenases
Source: Angew Chem Int Ed Engl. 2019 Oct 25;58(51):18710–4. doi: 10.1002/anie.201908258 (PMC6916344; doi:10.1002/anie.201908258)
Supplement: Supplementary file 1 — Supplementary [file ANIE-58-18710-s001.pdf]

Angewandte Chemie
Eine Zeitschrift der Gesellschaft Deutscher Chemiker

Supporting Information

X-ray Crystallography and Vibrational Spectroscopy Reveal the Key Determinants of Biocatalytic Dihydrogen Cycling by [NiFe] Hydrogenases

Yulia Ilina[+], Christian Lorent[+], Sagie Katz, Jae-Hun Jeoung, Seigo Shima, Marius Horch, Ingo Zebger,* and Holger Dobbek**

anie_201908258_sm_miscellaneous_information.pdf

Supporting Information

Table of Contents

Experimental procedure (p. 2)
Figure S1: Protomer and dodecamer composition of *Mb*FRH (p. 4)
Figure S2: Structure of the bridging [2Fe2S] cluster (p. 5)
Figure S3: Mononuclear Fe site (p. 6)
Figure S4: EPR spectrum recorded at 20 K (p. 7)
Figure S5: RR spectra of an *Mb*FRH single crystal recorded at different excitation wavelengths (p. 8)
Figure S6: Refinement of the [NiFe] active site after CO inhibition (p. 9)
Figure S7: Hydrophobic gas channel (p. 10)
Table S1: Diffraction and refinement statistics (p. 11)
Table S2: Various metal ions in the proximity to the [NiFe] active site (p. 12)
References (p. 13)
Author contributions (p. 13)

Experimental Procedures

All steps (cell growth, preparation of the cell extract, protein purification, characterization, and crystallization) were carried out under anoxic conditions, unless specified otherwise. All buffers were repeatedly degassed by evacuation and flushed with N_2. All protein depictions were prepared with Pymol and Chimera.[1]

Cultivation of *M. barkeri* MS. *Methanosarcina barkeri* MS (DSM 800) was obtained from the Deutsche Sammlung von Mikroorganismen und Zellkulturen (DSMZ, Braunschweig, Germany) as an actively growing culture. The archaeon was grown at 37 °C in 2-L -Schott glass bottles on methanol as a carbon and energy source. The growth medium, trace elements, and the vitamin solution were prepared according to Hippe et al.[2] with the following modification: 50 mM Bis-Tris buffer, pH 6.8, was added to the growth medium prior to sterilization. Cells reached the late exponential phase after ca. 55 h and were harvested by centrifugation in 1-L polycarbonate tubes with cap assembly. The cell pellet was flash-frozen in liquid N_2 and subsequently stored at -80 °C.

Purification of *Mb*FRH. Protein purification was performed at room temperature in an anoxic glove box (model B; COY Laboratory Products). 10 g of frozen cells were resuspended in 50 mL buffer A (25 mM HEPES, pH 7.5, 150 mM NaCl, 7.5 % (w/v) glycerol). The cell suspension was sonicated for 15 min (Bandelin Sonoplus 2200, 5 cycles, 50 % amplitude) in a rosette cooling cell on ice. After adding a small amount of DNase to the cell suspension, sonicated cells were centrifuged in a 60-mL gastight polycarbonate tube at 120,000 × g for 45 mins at 12 °C. The supernatant was concentrated to a volume of 20 mL (Amicon stirred cells with 500 kDa cut-off) and applied to a Sepharose 6B-CL column (ca. 400 mL bed volume, GE Healthcare Life Science) at 2 mL/min. Fractions of 10 mL volumes were collected, and those showing the highest rate of H_2-dependent reduction of oxidized methyl viologen were pooled and concentrated to ca. 7 mg/mL. Based on SDS-PAGE analysis, this sample contained around 40 % of the protein of interest and is denoted as hydrogenase-enriched sample in the following.

H_2-dependent reduction of oxidized methyl viologen. H_2-dependent reduction activity was assayed at room temperature by using oxidized methyl viologen (MV) as the electron acceptor. For the assay, 1 mL H_2-saturated buffer containing 50 mM HEPES-NaOH, pH 7.5, 100 mM NaCl, and 20 mM MV was placed in a screw-cap quartz cuvette sealed with a rubber septum. The cuvette was flushed with N_2 prior to usage. Reactions were initiated by injecting hydrogenase-enriched sample with a Hamilton syringe and monitored by the increase of absorbance at 578 nm (Agilent 8453 photodiode array spectrophotometer).

Crystallization. Protein crystallization was performed at 17 °C by the hanging-drop vapor diffusion method using 24-well plates. Crystals were grown in 0.1 M Tris-HCl, pH 7.8–8.5, 15–40 % (vol/vol) MPD by mixing 2.0 µL of 7 mg/mL hydrogenase-enriched sample with 2.0 µL of the reservoir solution. Crystals appeared within 2 to 4 days and were flash-frozen in liquid N_2 using 15 % (vol/vol) 2,3-butanediol as a cryoprotectant.

Xenon and carbon monoxide derivatization. *Mb*FRH crystals were exposed to 20–30 bar of Xe pressure for 15 min using a pressurized chamber at BESSY-II (Berlin, Germany). Crystal mounting and transfer into the pressurized chamber was done aerobically. For CO derivatization, *Mb*FRH crystals were transferred to a 48-well sitting-drop crystallization plate (Swissci 48-well MRC Maxi Optimization plate, Molecular Dimensions) filled with 10 µL of reservoir solution per well and covered with Scotch tape to prevent drying out. The plate was placed inside a self-made gastight chamber (Plexiglas, dimensions 15 cm × 15 cm × 5 cm) and incubated with 100 % CO under 1.5 bar for 30 h in the dark. Crystal harvesting was performed as described above for the as-isolated state.

Data collection, phasing, and structure refinement. Native and anomalous X-ray diffraction data were collected at 100 K at beam line BL 14.1 at BESSY-II. To confirm the positions of Fe ions, a dataset was collected at the high-energy side (λ = 1.732 Å) of the K-absorption edge of Fe to calculate an anomalous difference Fourier map. To reveal S and Xe positions, an X-ray wavelength of 1.900 Å was applied. Diffraction datasets were integrated and scaled with the X-ray Detector Software (XDS *via* the GUI XDSAPP).[3] Initial phasing was performed by molecular replacement with Phaser[4] using the homologous structure of *Mm*FRH (PDB entry: 4OMF).[5] Iterative cycles of model building and refinements were carried out with COOT.[6] Positional and temperature factor refinements were carried out with Refmac5[7] or Phenix.[8] Data statistics and final refinement statistics are reported in Table S1.

Electron paramagnetic resonance spectroscopy. 100-µL aliquots of protein solution with a concentration of ca. 50 µM were transferred into quartz electron paramagnetic resonance (EPR) tubes under anoxic conditions (5 % H_2 and 95 % N_2), precooled in cold ethanol (210 K), and stored in liquid N_2 (77 K). EPR measurements were performed on a X-Band continuous wave Bruker EMXplus spectrometer with an installed ER 4122 SHQE resonator. For temperature control, an Oxford EPR 900 He-flow cryostat equipped with an Oxford ITC4 controller was used in all experiments. The baseline of the obtained spectra was corrected by subtracting a background spectrum from buffer solution measured under the same experimental conditions. Experimental parameters: microwave power: 1 mW, microwave frequency: 9.3 GHz, modulation amplitude: 10 G, modulation frequency: 100 kHz.

Infrared and resonance Raman spectroscopy. Several single crystals of *Mb*FRH, grown under anoxic conditions (5 % H_2 and 95 % N_2) and taken from the same batch the crystal structure was derived from, were collected, placed on homemade MgF_2 optical plates, and subsequently frozen in liquid N_2 for further analysis by infrared (IR) and resonance Raman (RR) spectroscopy. Single crystals incubated with 100 % CO were prepared the same way.

IR spectra with a spectral resolution of 2 cm^{-1} were recorded using a Bruker Tensor 27 FT-IR spectrometer linked to a Hyperion 3000 IR microscope equipped with a 20× IR transmission objective and a mercury cadmium telluride (MCT) detector. Throughout the measurements, the sample temperature was set to 80 K *via* a liquid-N_2-cooled cryo-stage (Linkam Scientific Instruments). The presented final spectra represent an average of ten datasets each composed of 200 interferometer scans. For data recording and evaluation, the Bruker OPUS software version 6.5 or higher was used.

RR spectra were obtained using a LabRam HR-800 Jobin Yvon confocal Raman spectrometer connected to a liquid-N_2-cooled charge-coupled device (CCD). Measurements were performed at 80 K using a liquid-N_2-cooled cryo-stage (Linkam Scientific instruments). Kr^+ or Ar^+ ion laser beams were focused at a 2–4 µm spot on the surface of the single crystal with an overall beam power of 1–2 mW. The displayed RR spectra represent an average of 30–50 individual spectra, each accumulated for 180–300 s. Toluene was used as an external standard for frequency calibration of the spectra.

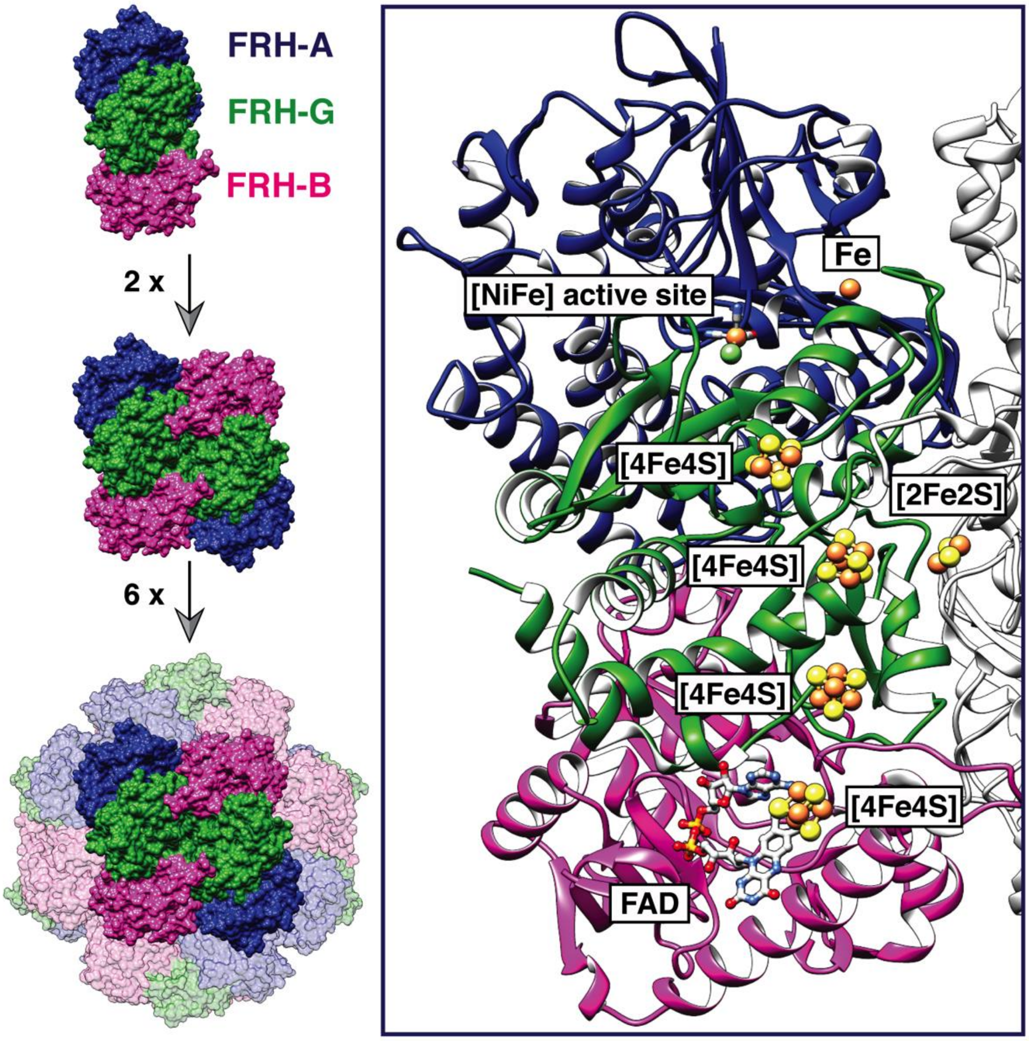

Figure S1. The protomer of the *Mb*FRH is a heterotrimer, consisting of the subunits FRH-A (blue), FRH-B (violet) and FRH-G (green), represented as surfaces or ribbons. From these three subunits, the crystallographic symmetry creates a dodecameric arrangement with the shape of a spherical ball with a diameter of 190 Å and a molecular weight of 1.25 MDa, which is consistent with the mass found for the homologous enzymes from *M. marburgensis*,[5] *M. thermoautotrophicus,*[9] *M. voltae,*[10] *M. jannaschii* [11] and *M. formicicum*.[12] FeS clusters, a mononuclear Fe center, and the [NiFe] active site are shown as spheres (yellow for S, orange for Fe, and green for Ni). The FAD molecule is shown in form of sticks.

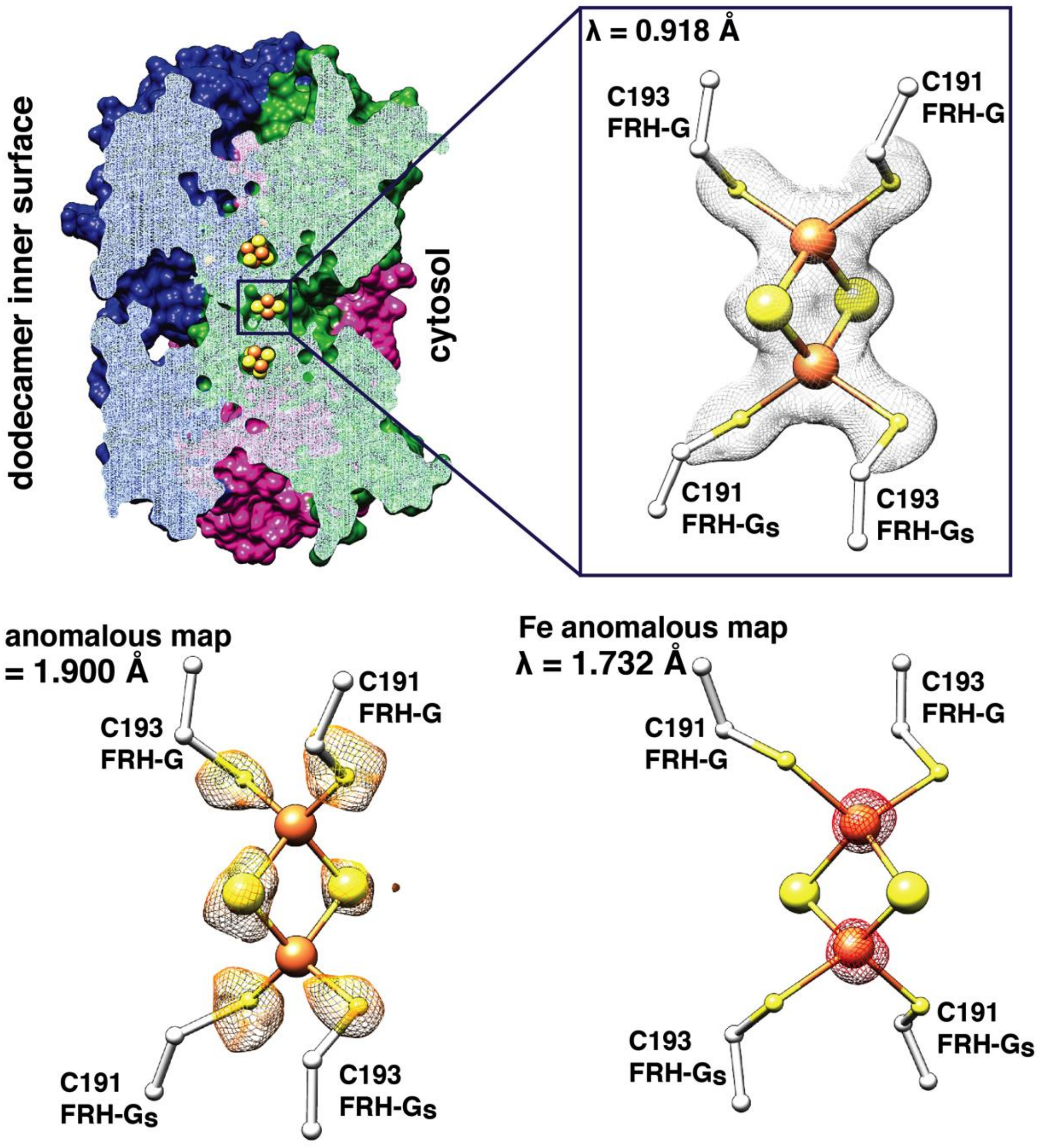

Figure S2. The solvent-accessible [2Fe2S] cluster is cysteine-coordinated and bound between two FRH-G subunits at the two-fold symmetry axis of FRH protomers. Fe (yellow) and S (orange) atoms are displayed as spheres. Amino acid residues of the different FRH-G subunits (denoted as FRH-G and FRH-G_S) are shown as sticks. The electron density maps are depicted as meshes ($2F_{obs}-F_{calc}$ after full refinement in gray (1.8 σ), Fe anomalous in orange (7.0 σ), S anomalous in yellow (3.0 σ)). FRH-A (blue), FRH-B (violet), and FRH-G (green) subunits are represented as surfaces.

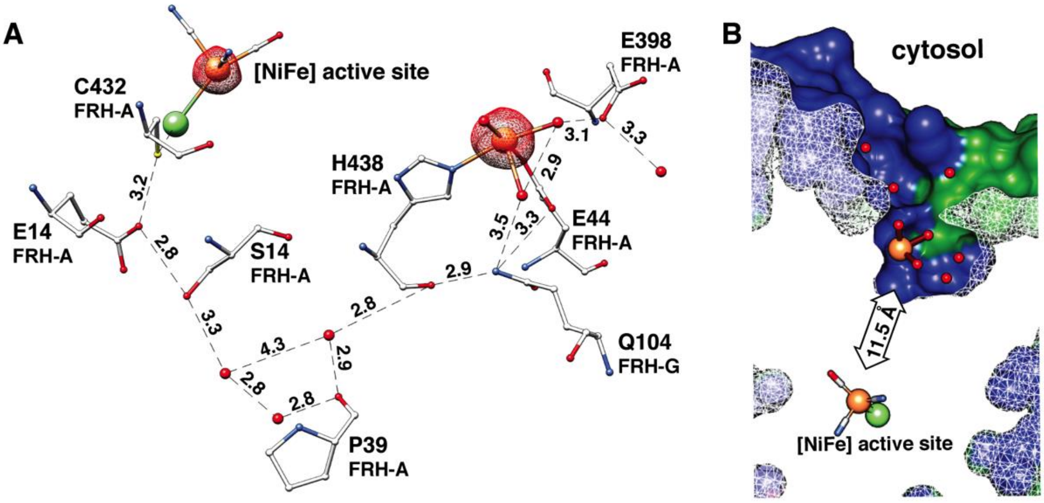

Fig. S3. (A) The single Fe ion is octahedrally coordinated by the side chains of Glu44 and His438, the carbonyl-oxygen of Met399 (not shown), and three water molecules. (B) It is located within a hydrophilic channel approximately 11.5 Å from the active site and could play a role in one of the predicted proton transport pathways in group 1 [NiFe] hydrogenases.[13] The Fe anomalous map is shown as a red mesh (8.0 σ). Distances are stated in Å. Metal ions and amino acids are represented as spheres (green for Ni and orange for Fe) and sticks, respectively.

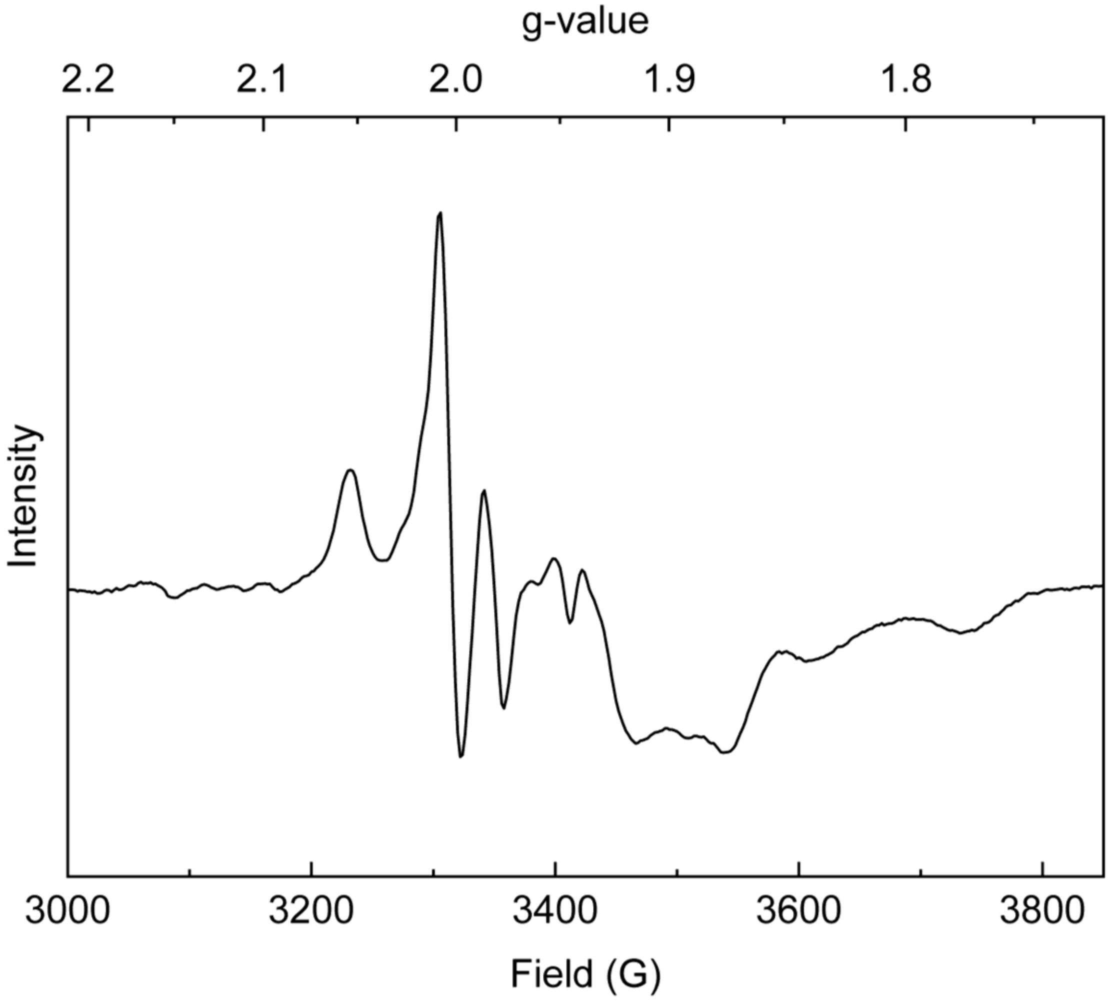

Figure S4. EPR spectrum of *Mb*FRH solution, recorded at 20 K with 1 mW microwave power and 9.3 GHz microwave frequency. Detected signals correspond to FAD, [2Fe2S] clusters, and [4Fe4S] clusters. The latter of the metal centers are usually not detected at higher temperatures (above 30 K) due to their faster spin relaxation. No substantial signals corresponding to EPR-active states of Ni have been observed.

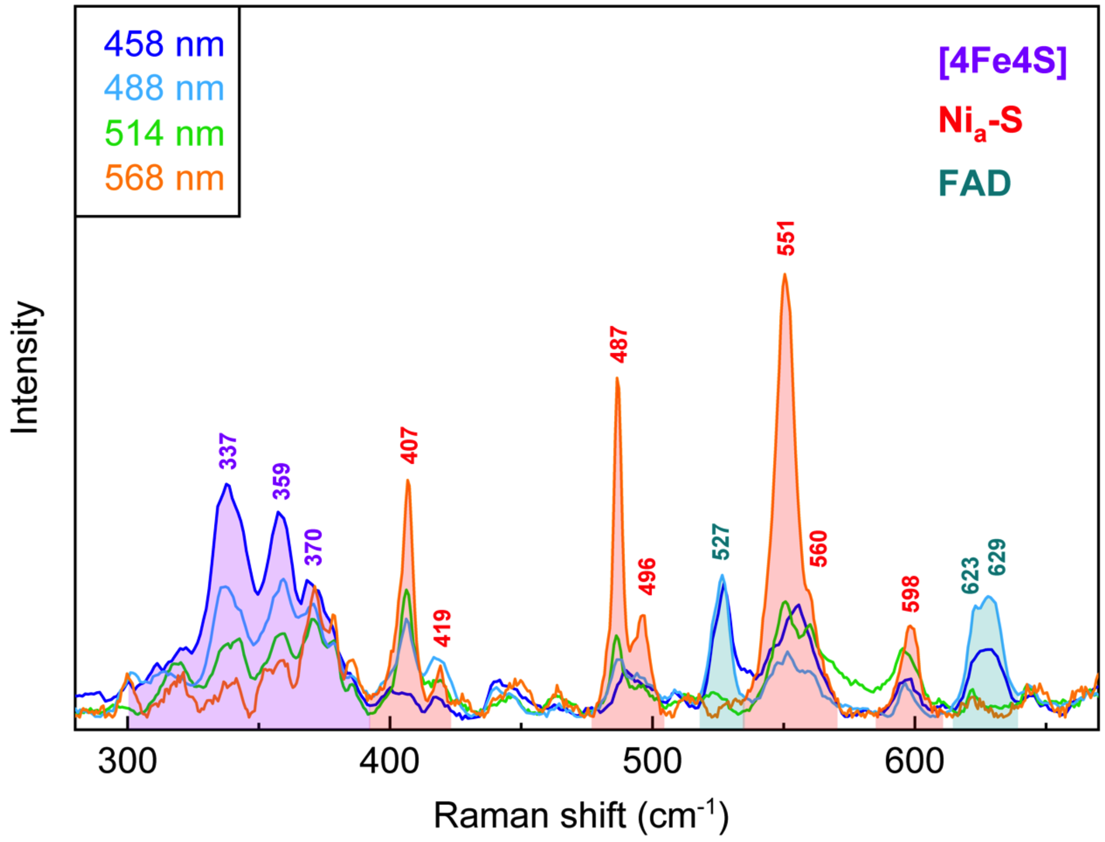

Figure S5. Signals from [4Fe4S] clusters are labeled in purple, while contributions from FAD and the [NiFe] active site in the Ni$_a$-S state are marked in dark cyan and red, respectively. Spectra were normalized with respect to the intensity of a non-resonant phenylalanine marker band at 1004 cm^{-1}.[14]

A

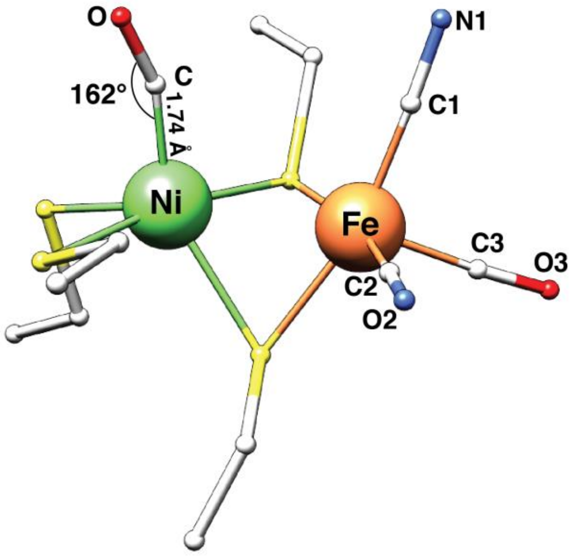

B

Atom	Occupancy	Temp. factor
N2	1.0	33.79
C2	1.0	31.78
FE	1.0	33.25
C3	0.95	37.61
O3	0.9	35.06
C1	0.98	31.13
N1	1.0	31.68
NI	1.0	37.44
C	0.52	36.01
O	0.49	36.47

Figure S6. (A) In the Ni_a-S state, CO binds specifically to the Ni atom of the [NiFe] active site with a Ni–C distance of 1.74 Å and a Ni–C–O angle of 162°. Amino acids and metals are depicted as sticks and spheres, respectively. (B) The CO ligand was refined to 50 % occupancy. Chemical restrain parameters for the CO binding were generated by iterative cycles of refinement based on the homologous models of CO-inhibited [NiFe] hydrogenase from *Desulfovibrio vulgaris* Miyazaki F.[15]

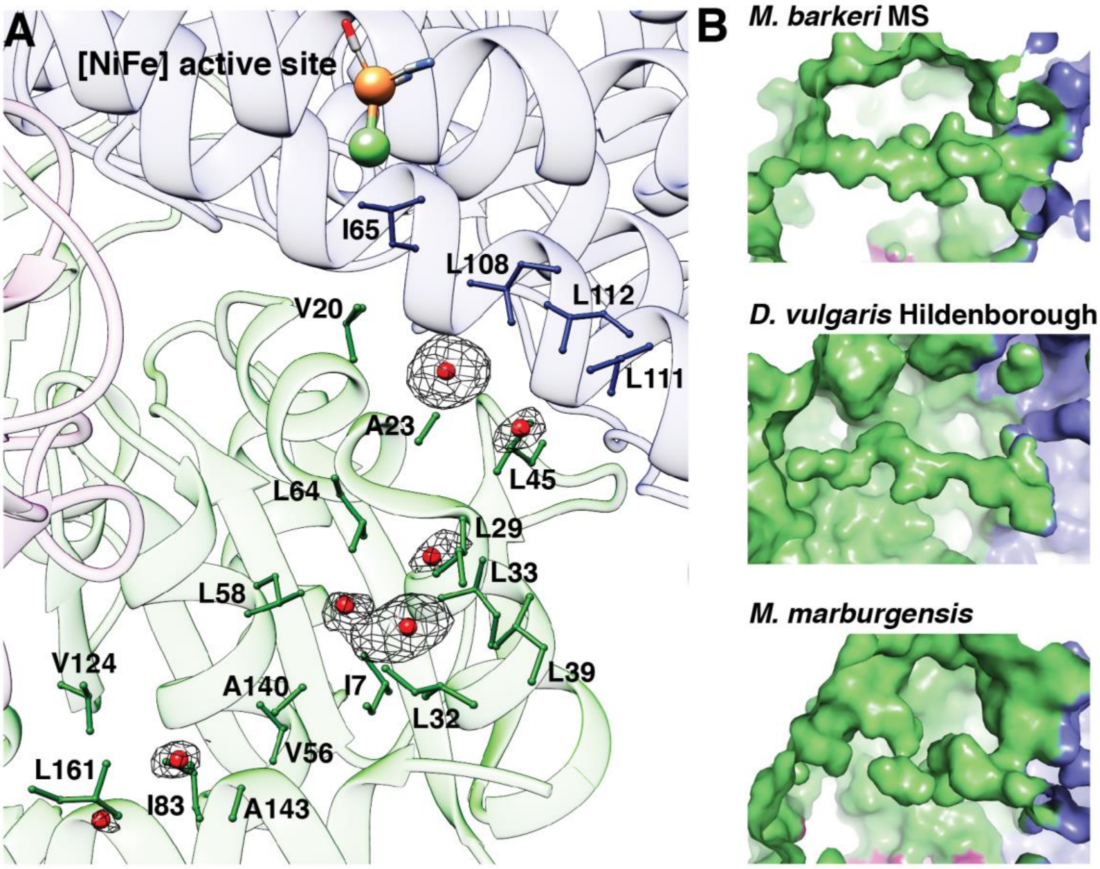

Figure S7. (A) Side chains of channel-lining amino acids (in one-letter code) forming a noncanonical hydrophobic channel of *Mb*FRH are shown as sticks. Seven Xe atoms (red spheres) were detected (anomalous map as black mesh at 3.0 σ). (B) Comparison of [NiFe] hydrogenases revealed the presence of a similar noncanonical channel in [NiFeSe] hydrogenase from *Desulfovibrio vulgaris* Hildenborough (PDB entry: 3ZEA), whereas in *Mm*FRH such a channel may be interrupted (PDB entry: 4OMF). FRH-A (and large subunit of the bacterial homologue), FRH-B, and FRH-G (and small subunit of the bacterial homologue) are represented as ribbons or surfaces and colored as navy blue, violet, and green respectively.

	“as-isolated”[1] (native)	“as-isolated” (Fe set)	Xe derivatization[2] and S set	CO derivatization[3]
**Data collection**				
Wavelength (Å)	0.918	1.732	1.900	0.918
Resolution range (Å)[4]	50 - 1.84 (1.95 - 1.84)	50 - 2.06 (2.18 - 2.06)	50 - 2.28 (2.42 - 2.28)	50 – 1.99 (2.11 – 1.99)
Space group	*F*23	*F*23	*F*23	*F*23
Cell dimensions a=b=c (Å) α=ß=γ (°)	235.86 90	236.33 90	236.82 90	235.43 90
Completeness (%)	99.6 (99.3)	99.5 (97.8)	99.8 (99.9)	99.9 (99.3)
Observed/unique reflections	744324 / 183316 (114544 / 29569)	643642 / 131296 (83050 / 20763)	1978852 / 97909 (300327 / 15842)	1512394 / 73859 (236179 / 11826)
$R_{obs.}$ (%)	18.3 (211.5)	10 (176.7)	9.0 (149.4)	23.0 (256.5)
$R_{meas.}$ (%)	21.1 (243.9)	11.2 (201.9)	9.3 (153.5)	23.6 (263.2)
I / σI	6.62 (0.58)	10.51 (0.63)	23.32 (1.76)	12.73 (1.22)
CC (1/2)	99.3 (20.9)	99.7 (26.7)	99.9 (76.3)	99.8 (53.9)
**Refinement**				
R_{work} / R_{free} (%)	0.1753 / 0.2206	0.1935 / 0.2266	0.1760 / 0.2270	0.1688 / 0.2092
Molecules in ASU	1	1	1	1
Ramachandran statistics (%) preferred / allowed / disallowed	97.13 / 2.67 / 0.21	96.1 / 3.1 / 0.8	95.08 / 4.72 / 0.21	97 02 / 2.87 / 0.1
Rms deviation from ideal geometry<br>Bond lengths (Å)<br>Bond angles (°)	 0.017<br>1.337	 0.0193<br>1.8587	 0.011<br>1.143	 0.013<br>1.169

Table S1. Diffraction and refinement statistics.

[1] PDB entry: 6QGR
[2] PDB entry: 6QII
[3] PDB entry: 6QGT
[4] Values in parentheses for highest resolution shell

Table S2: In [NiFe] hydrogenases, the position of the mononuclear metal atom is occupied with diverse bivalent ions, where coordinating residues are highly conserved among different species. LS stands for the large subunit of group 1 hydrogenases, which is homologous to the FRH-A subunit of archaeal F_{420}-reducing group 3 [NiFe] hydrogenases.

<table>
<tr><th></th><th></th><th colspan="3">Metal-coordinating residues</th><th colspan="2">Water-interacting residues</th><th></th></tr>
<tr><td rowspan="2">[NiFe] group 3</td><td>*M. barkeri* MS</td><td>H438 FRH-A</td><td>M399 FRH-A</td><td>E44 FRH-A</td><td>H105 FRH-G</td><td>E259 FRH-A</td><td>Fe^{2+}</td></tr>
<tr><td>*M. methanotermobacter*</td><td>His386 FRH-A</td><td>A347 FRH-A</td><td>E44 FRH-A</td><td>T248 FRH-G</td><td>E229 FRH-A</td><td rowspan="3">Mg^{2+}</td></tr>
<tr><td rowspan="3">[NiFe] group 1</td><td>*D. fructosovorans*</td><td>H549 LS</td><td>L495 LS</td><td>E53 LS</td><td>K372 LS</td><td>E334 LS</td></tr>
<tr><td>*E. coli*</td><td>H582 LS</td><td>C528 LS</td><td>E57 LS</td><td>K399 LS</td><td>E347 LS</td></tr>
<tr><td>*M. thermolithotrophicus*</td><td>H448 LS</td><td>L395 LS</td><td>E42 LS</td><td>K273 LS</td><td>E264 LS</td><td>Ca^{2+}</td></tr>
<tr><td rowspan="2">[NiFeSe] group 1</td><td>*D. baculatum*</td><td>H498 LS</td><td>I444 LS</td><td>E51 LS</td><td>K321 LS</td><td>E285 LS</td><td rowspan="2">Fe^{2+}</td></tr>
<tr><td>*D. vulgaris* Hildenborough</td><td>H495 LS</td><td>I441 LS</td><td>E56 LS</td><td>K317 LS</td><td>E282 LS</td></tr>
</table>

References

[1] E. F. Pettersen, T. D. Goddard, C. C. Huang, G. S. Couch, D. M. Greenblatt, E. C. Meng, T. E. Ferrin, J Comput Chem 2004, 25, 1605-1612.
[2] H. Hippe, D. Caspari, K. Fiebig, G. Gottschalk, Proc Natl Acad Sci U S A 1979, 76, 494-498.
[3] W. Kabsch, Acta Crystallogr D Biol Crystallogr 2010, 66, 125-132.
[4] P. H. Zwart, P. V. Afonine, R. W. Grosse-Kunstleve, L. W. Hung, T. R. Ioerger, A. J. McCoy, E. McKee, N. W. Moriarty, R. J. Read, J. C. Sacchettini, N. K. Sauter, L. C. Storoni, T. C. Terwilliger, P. D. Adams, Methods Mol Biol 2008, 426, 419-435.
[5] S. Vitt, K. Ma, E. Warkentin, J. Moll, A. J. Pierik, S. Shima, U. Ermler, J Mol Biol 2014, 426, 2813-2826.
[6] P. Emsley, K. Cowtan, Acta Crystallogr D Biol Crystallogr 2004, 60, 2126-2132.
[7] G. N. Murshudov, P. Skubak, A. A. Lebedev, N. S. Pannu, R. A. Steiner, R. A. Nicholls, M. D. Winn, F. Long, A. A. Vagin, Acta Crystallogr D Biol Crystallogr 2011, 67, 355-367.
[8] P. D. Adams, P. V. Afonine, G. Bunkoczi, V. B. Chen, I. W. Davis, N. Echols, J. J. Headd, L. W. Hung, G. J. Kapral, R. W. Grosse-Kunstleve, A. J. McCoy, N. W. Moriarty, R. Oeffner, R. J. Read, D. C. Richardson, J. S. Richardson, T. C. Terwilliger, P. H. Zwart, Acta Crystallogr D Biol Crystallogr 2010, 66, 213-221.
[9] J. A. Fox, D. J. Livingston, W. H. Orme-Johnson, C. T. Walsh, Biochemistry 1987, 26, 4219-4227.
[10] E. Muth, E. Morschel, A. Klein, Eur J Biochem 1987, 169, 571-577.
[11] N. N. Shah, D. S. Clark, Appllied and Environmental Microbiology 1990, 56, 858-863.
[12] S. F. Baron, J. G. Ferry, JOURNAL OF BACTERIOLOGY 1989, 171, 3846-3853.
[13] H. Ogata, W. Lubitz, Y. Higuchi, Dalton Trans 2009, 7577-7587.
[14] aM. Horch, J. Schoknecht, M. A. Mroginski, O. Lenz, P. Hildebrandt, I. Zebger, J Am Chem Soc 2014, 136, 9870-9873; bJ. P. M. Schelvis, D. Pun, N. Goyal, O. Sokolova, Journal of Raman Spectroscopy 2006, 37, 822–829; cS. Todorovic, M. Teixeira, J Biol Inorg Chem 2018, 23, 647-661.
[15] H. Ogata, Y. Mizoguchi, N. Mizuno, K. Miki, S. Adachi, N. Yasuoka, T. Yagi, O. Yamauchi, S. Hirota, Y. Higuchi, J Am Chem Soc 2002, 124, 11628-11635.

Author Contributions

Yulia Ilina (equal): experimental implementation, data curation, analysis and validation of the X-Ray crystallographic part as well as writing of the original draft
Christian Lorent (equal): experimental implementation, data curation, analysis and validation of the vibrational and electron paramagnetic resonance spectroscopic part as well as writing of the original draft
Dr. Marius Horch (supporting/lead); data curation, analysis and validation, project administration as well as writing of the original draft
Dr. Ingo Zebger (supporting/lead): funding acquisition, data analysis, validation and project administration, contributions to original draft
Prof. Dr. Holger Dobbek (supporting/lead): funding acquisition, data analysis, validation, project administration and writing of the original draft
Dr. Jae-Hun Jeoung (supporting): suggestions regarding X-ray data analysis and validation as well as experimental design
Dr. Sagie Katz (supporting): initial IR spectroscopic experiments, data curation, analysis and validation
Dr. Seigo Shima (supporting): support on cultivation of archaea
